# The Impact of Positive Fluid Balance on Sepsis Subtypes: A Causal Inference Study

**DOI:** 10.1155/2023/2081588

**Published:** 2023-10-03

**Authors:** Sharad Patel, Adam Green, Yanika Wolfe, Gregory Felock, Samantha Epstein, Nitin Puri

**Affiliations:** Cooper University Hospital, Camden, 1 Cooper Plaza, NJ 08103, USA

## Abstract

**Introduction:**

Sepsis, the leading cause of death in hospitalized patients globally, was investigated in this study, examining the varying effects of positive fluid balance on sepsis subtypes through causal inference.

**Methods:**

In this study, data from the eICU database were utilized, extracting 35 features from sepsis patients. Fluid balance during ICU stay was the treatment, and ICU mortality was the primary outcome. Data preprocessing ensured linear assumptions for logistic regression. Binarized positive fluid balance with mortality was examined using DoWhy's logistic regression, while continuous data were analyzed with random forest T-learner. ATE served as the primary metric.

**Results:**

Results revealed that septic patients with higher fluid balance had worse mortality outcomes, with an ATE of 0.042 (95% CI: (0.034, 0.047)) using logistic regression and an ATE of 0.0340 (95% CI: (0.028–0.040)) using T-learner. In the pulmonary sepsis subtype, higher mortality was associated with increased fluid balance, showing an ATE of 0.047 (95% CI: (0.037, 0.055)) using logistic regression and an ATE of 0.28 (95% CI: (0.22, 0.34)) with T-learner. Conversely, urinary sepsis patients had improved mortality with higher fluid balance, presenting an ATE of −0.135 (95% CI: (−0.024, −0.0035)) using logistic regression and an ATE of −0.28 (95% CI: (−0.34, −0.22)) with T-learner.

**Conclusion:**

Our research implies that fluid balance impact on ICU mortality differs among sepsis subtypes. Positive fluid balance raises mortality in sepsis and pulmonary sepsis but may protect against urinary sepsis. Further trials are needed to confirm these findings.

## 1. Introduction

Sepsis is a dysregulated host immune response to active infection that can lead to end-organ damage. It is a leading cause of death in hospitalized patients, with an estimated 28.9 million cases and 6.3 million deaths worldwide each year [1]. Although thoroughly studied, a few novel treatments have been developed over the past few decades. Sepsis is a complex and multifaceted syndrome, with variations in the causative pathogen, site of infection, and degree of dysregulated host response [2]. Studying a heterogeneous disorder, such as sepsis, with the assumption of uniformity reduces the probability of identifying treatments that may be promising for larger subtypes [3]. This may explain why many therapies with biological plausibility do not translate into clinical results. The challenges of designing sepsis trials may be attributed to their heterogeneity, underscoring the need to identify specific subtypes or “phenotypes” of the disease that may benefit from targeted treatments. Identifying these sepsis phenotypes may improve our understanding of the underlying mechanisms of the disease and enhance the design of clinical trials to achieve more successful treatment outcomes. Recent studies using unsupervised machine learning have shown promising results in characterizing sepsis phenotypes, offering hope for the future of more effective sepsis trial design and treatment [4].

**Figure 1:**
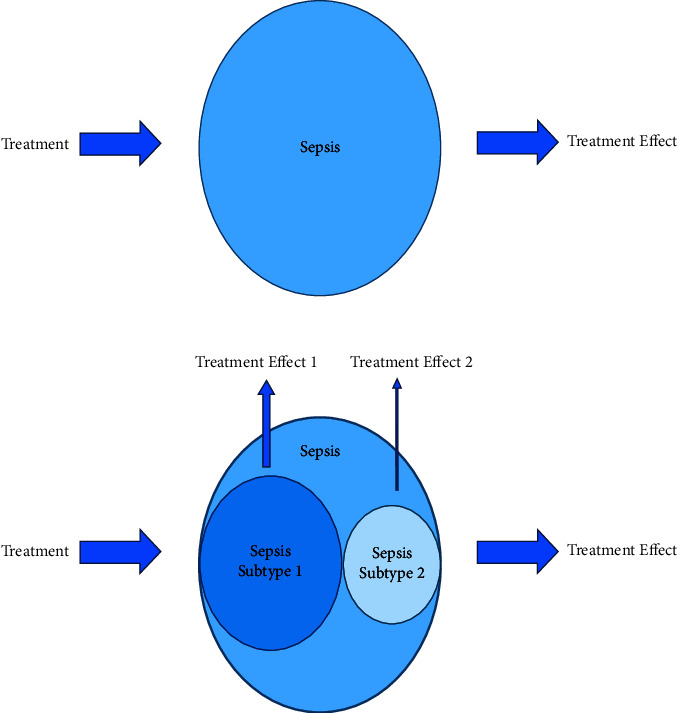
The current approach to studying sepsis treatment that diminishes the heterogeneity of the syndrome. The second image provides a more nuanced approach as it separates large subtypes within sepsis and may allow for more individualized treatment discovery.

The goal of fluid resuscitation in sepsis treatment is to restore blood pressure and improve organ perfusion [5, 6]. However, recent studies have shown that excessive or prolonged fluid administration can increase mortality [7, 8]. Nevertheless, it is plausible that some sepsis subtypes may benefit from positive fluid balance, while others may be harmed. Fluid administration based on sepsis subtypes has not yet been investigated.  Figure 1 illustrates the current method for studying sepsis as a monolithic entity. However, we propose that larger subtypes should be studied separately, given the heterogeneity of sepsis, with the hope of providing individualized and precision medicine.

Randomized control trials remain the gold standard for estimating treatment effects, as this limits the number of confounders. However, they are resource-laden and expensive and can have methodological problems. Therefore, there has been increased interest in statistical and machine-learning methods to estimate causal effects called causal inference [9, 10]. Statistical causal inference determines the causal effect of treatment or exposure on an outcome by using statistical methods. An example of a statistical method used in causal inference is inverse probability of treatment weighting (IPTW). IPTW uses the probability of receiving treatment to balance the baseline characteristics between the treated and untreated groups, thereby allowing for a more accurate estimate of the causal effect of the treatment on the outcome [11]. Using causal inference, we aimed to retrospectively discern the varying effects of positive fluid balance on sepsis subtypes. We employed a simple mental model to separate sepsis subtypes according to the site of infection. We hypothesized that positive fluid balance will negatively impact sepsis mortality.

## 2. Methods

### 2.1. Ethics Statement

This study analyzed a publicly available anonymized database with approval from a preexisting institutional review board.

### 2.2. Sample Selection

**Figure 2:**
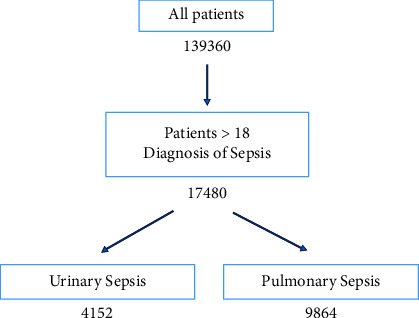
A total of 139,360 patients were available in the eICU database, out of which 17,480 had an initial diagnosis of sepsis and greater than 18 years of age. The two largest subgroups were identified as pulmonary and urinary sepsis.

The eICU Collaborative Research Database is a multicenter intensive care unit database with data from over 200,000 intensive care unit (ICU) admissions monitored by eICU programs [12]. The eICU database comprises 200,859 patient unit encounters for 139,367 unique patients admitted between 2014 and 2015 from 208 hospitals located throughout the US. Adult patients were included in eICU (Figure 2). In addition, patients diagnosed with urinary or pulmonary sepsis were queried from the dataset. 35 features were extracted from the eICU database. The primary outcome was ICU mortality, whereas the primary treatment was the total fluid balance in the ICU.

Feature sets with more than 40 percent missing values were dropped, and scales and transformations were applied to meet the linear assumptions for logistic regression [1]. Based on the initial visual analysis of outliers in the fluid balance feature using violin plots, we excluded patients with fluid balance greater than 15 and less than –15 liters. The Sklearn robust scaler was used [13]. The data remained highly skewed; therefore, further transformation using the Yeo–Johnson transformer was utilized [13]. Outliers were removed to reduce the skew for all features to be less than 0.5 and greater than −0.5. The three sepsis groups were compared in this analysis. The first group comprised the entire sepsis cohort, which was compared with the pulmonary and urinary sepsis groups. Our primary analysis method was logistic regression in the DoWhy library, which requires binarization of treatment features. The net fluid balance of each sepsis subtype was binarized using the SKlearn binarizer with the mean (after initial feature transformation to create normal distribution) of the features as the cutoff. Using logistic regression as the primary model and AUC as the metric, recursive feature selection was performed using the library from the feature engine.

**Figure 3:**
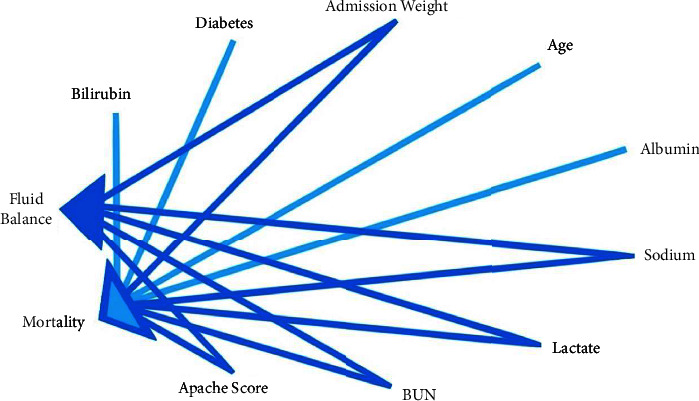
A directed acyclic graph (DAG) is a graphical representation of a set of variables and the relationships between them. Effect modifiers (light blue) and confounders (blue) are two important concepts in causal inference. Effect modifiers are variables that modify the effect of a treatment or exposure on an outcome. Confounders, on the other hand, are variables that are associated with both exposure and outcome and thus may bias estimates of the treatment effect.

We utilized the DoWhy library in Python to perform causal inference on the data in four steps: model, identify, estimate, and refute [14]. Our primary metric for evaluating the degree of causality is the average treatment effect (ATE). In the model step, we utilized the domain expertise of the four intensivists to identify confounders and effect modifiers to construct a formal causal model. First, using domain expertise for our directed acyclic graph, nine features were identified as effect modifiers and confounders (Figure 3). Second, the causal estimate was determined, which in our analysis was noted to be the back-door criterion [9]. Third, the causal effect was derived via inverse propensity weighting with logistic regression or a metalearner via a random forest model [15, 16]. Finally, multiple methods refute the estimate by adding a random common cause and data subset analysis, assuming that ATE should not vary significantly from the original values if our results are valid.

Our primary model used to infer ATE for the binarized treatment was inverse probability weighting (IPTW) with logistic regression. IPTW is a method used to estimate the causal effect of a binary treatment on an outcome variable. It involves weighting each individual in the sample according to the inverse of their probability of receiving the treatment and then estimating the treatment effect using a weighted regression model.

We further validated our findings by analyzing the continuous treatment values of positive fluid balance using a machine-learning method called T-learners from the EconMl library [17]. T-learners combined a treatment assignment and response model to calculate the causal effects of treatment on outcomes. It can be implemented using various machine-learning algorithms, including the random forest model. Random forest is a machine-learning algorithm that creates a collection of decision trees and uses them to make predictions. One of the strengths of this method is its ability to handle both continuous and binary treatments. Considering the robustness of tree-based models to outliers and scales, minimal preprocessing of the data was performed for learner analysis beyond initial outlier removal using visual inspection.

## 3. Results

This study aimed to evaluate the causal effect of fluid balance on ICU mortality in a sample of patients admitted to the ICU. Summary statistics stratified by mortality are presented in Table 1, which demonstrates a statistically significant difference in all features between alive and expired patients. The mortality outcome displayed a marked imbalance, with only ten percent of the patients having died. The results showed that expired patients had a higher mean fluid balance than the live cohort.

To estimate the causal effect of fluid balance on mortality, we utilized two models: an IPTW logistic regression model with binarized treatment and a random forest T-learner model with continuous treatment. ATE was calculated for sepsis subtypes (Table 2). The results of our study with the logistic regression/binarized analysis showed that sepsis overall had worse mortality outcomes, with an ATE of 0.042 (95% CI: (0.034, 0.047)). In addition, the T-learner model that utilized continuous fluid balance values demonstrated an ATE of 0.034 (95% CI: (0.028, 0.040)). This suggests that patients with sepsis who received higher fluid balance had worse mortality outcomes.

When looking at specific subtypes of sepsis, pulmonary sepsis had a worse outcome than the total sepsis group, with an ATE of 0.047 (95% CI: (0.037, 0.055)) in the IPTW model. The T-learner model demonstrated a large effect with an ATE of 0.28 (95% CI: (0.22, 0.34)). This suggests that patients with pulmonary sepsis who received higher fluid balance had worse mortality outcomes. In contrast, urinary sepsis had improved outcomes with positive fluid balance and an ATE of −0.135 (95% CI: (−0.024, −0.0035)). The T-learner model demonstrated a large mortality benefit with an ATE of −0.28 (95% CI: (−0.34, −0.22)). This suggests that patients with urinary sepsis who received higher fluid balance had better mortality outcomes.

Furthermore, we performed a refutation analysis using the DoWhy library. We chose two methods to perform refutation: adding a random confounder and utilizing only a subset of data. Refutation analysis in causal inference adds robustness to the results by challenging the validity of causal assumptions. The DoWhy library refutation method performs this by adding a random common cause or using a subset of data. In doing so, it assesses the sensitivity of the causal effect estimates to the choice of variables and data. If the causal effect estimates are robust to these challenges, confidence in the validity of the causal inference results is increased. Our refutation findings, summarized in Table 3, demonstrated minimal differences in ATE for both methods of refutation.

## 4. Discussion

Our study shows that there is indeed variation in ATEs among sepsis subtypes with positive fluid balance. We used causal inference techniques to discern the effect of positive fluid balance on sepsis subtypes. The results of our study indicate that positive fluid balance negatively impacts sepsis overall, but there was significant heterogeneity within the subtypes. The outcomes of pulmonary sepsis were significantly worsened by positive fluid balance, whereas those of urinary sepsis improved. With higher fluid balance, we hypothesized that pulmonary sepsis would have negative effects, but we expected urinary sepsis to also have negative effects, which was not the case. This relationship was consistent in both our binarized treatment IPTW model and the continuous treatment T-learner model.

Sepsis can take many different forms and is characterized by marked heterogeneity. One approach to understand the heterogeneity of sepsis is to classify patients into different phenotypes or subtypes based on their clinical characteristics and response to treatment [4]. Sepsis phenotypes can be defined based on various factors, including the underlying source of the infection, the presence of organ dysfunction, and degree of inflammation, to name a few. Prior studies have even phenotyped them based on genomics [18]. Our study employed a more simplistic but likely practical phenotyping of sepsis at the site of infection.

One possible mechanism for the negative effect of positive fluid balance in sepsis and pulmonary sepsis is that it may lead to fluid overload in the setting of known sepsis-related glycocalyx damage [19]. This is empirically supported by prior research, which suggests that fluid overload is associated with increased mortality in critically ill patients with sepsis [20–22]. Pulmonary sepsis appears to be especially sensitive to positive fluid balance, as demonstrated in prior studies [23]. Seethala et al. reported that patients with pneumonia as the primary site of sepsis had an odds ratio of 2.31 for progression to acute respiratory distress syndrome (ARDS) in the setting of positive fluid balance, and those that progressed to ARDS had higher mortality [24]. Patients with pulmonary sepsis may be at an increased risk of progression to ARDS because of positive fluid balance due to increased capillary permeability, leading to pulmonary edema. It is unclear why positive fluid balance appeared to have a protective effect on urinary sepsis in our study, but this finding needs to be validated prospectively.

One of the key strengths of this study is the use of causal inference techniques, specifically IPTW and T-learner models, to estimate the causal effect of fluid balance on ICU mortality in sepsis subtypes. We employed multiple models with both binary and continuous values, which we believe adds credibility to our assertions. Compared to traditional observational methods, this method provides a more robust and accurate estimate of causal effects. Our study also employed refutation analysis, which is a robust way to validate causal assumptions, adding further confidence in the validity of the results. In addition, although our study was retrospective, the sample size was relatively large. We believe that the simplicity of our phenotyping method is strength as it provides a simple mental model that can potentially be applied with relative ease. Our study adds to the current evidence by demonstrating that the effect of positive fluid balance on sepsis outcomes may vary depending on the subtype of sepsis, which, to the best of our knowledge, has not been studied. Our results suggest that positive fluid balance may harm patients with sepsis overall; some subtypes fare worse, while others have a beneficial effect. One potential implication of our findings may be the refinement of sepsis fluid expansion guidelines that account for the sepsis subtype as a factor in resuscitation. By phenotyping sepsis into subtypes based on the site of infection, our study provides a more nuanced understanding of the effects of fluid balance on sepsis outcomes.

A practical application of our findings is to adjust fluid resuscitation strategies in sepsis according to the suspected site of infection based on clinical presentation, cultures, and imaging. For example, a reduced initial fluid bolus could be considered for patients presenting with pulmonary sepsis as compared to urinary sepsis, given the increased risk of harm with positive fluid balance our study found in pulmonary sepsis. If the patient with pulmonary sepsis remains hypotensive after an initial conservative fluid bolus, earlier initiation of vasopressor therapy may be preferred over additional fluid boluses to avoid worsening pulmonary edema and progression to ARDS. In contrast, patients presenting with urinary sepsis may benefit from a more liberal initial fluid bolus if they remain hypotensive, given the potential mortality benefit seen with higher fluid balance in this subtype in our analysis. As additional microbiology and imaging data become available, the working diagnosis of the sepsis source may be refined, and fluid management adjusted accordingly. While our retrospective analysis provides a foundation, prospective clinical trials are needed to validate optimal individualized fluid strategies based on the sepsis source. Our study has several limitations. This was a retrospective analysis, and the conclusions must be validated prospectively.

We utilized causal inference techniques with the aim of implying causality, but the gold standard remains RCT. Our primary causal inference model can only handle binary treatments, but fluid balance data are continuous, which requires a significant amount of preprocessing and transformation with the possibility of information loss in these transformations. We supplemented the binarized analysis with concurrent analysis of the continuous data to allay this limitation. In addition, the T-learner model requires minimal preprocessing, reducing the concern that significant preprocessing required in the logistic regression model limits our inferences. The visual approach to outlier removal could introduce biases, but our intention was to avoid large impacts from extreme outliers. We mitigated this by also using the Sklearn robust scaler and Yeo–Johnson transformer to minimize the impact of extreme values. While choosing the mean as a threshold for binarization initially presents a limitation, we mitigated this effect by reducing the data skew to less than 0.5, ensuring a more symmetric distribution and enhancing the appropriateness of the mean as a representative measure of the central tendency for our analysis.

## 5. Conclusions

In conclusion, our study showed a variation in the effect of positive fluid balance on sepsis subtypes. We used causal inference techniques to estimate the causal effect of fluid balance on ICU mortality in sepsis subtypes and found that positive fluid balance negatively impacted sepsis overall but with significant heterogeneity within the subtypes. However, our study was retrospective, and the conclusions must be validated prospectively.

## Figures and Tables

**Table 1:** Summary statistics stratified by mortality, means ± SD, or *N* (%).

Features	Overall	Alive	Expired
*N*	17,480	15,699	1,781
Apache score	67.6 ± 26.3	64.6 ± 23.8	94.6 ± 31.5
Admission weight (kg)	81.4 ± 27.9	81.6 ± 28.0	79.6 ± 27.1^*∗∗*^
Sodium (mmol/L)	139.7 ± 5.9	139.6 ± 5.8	140.5 ± 6.9
Diabetes	13,352 (76.4%)	11,926 (89.3%)	1,426 (10.7%)
Bilirubin (mg/dL)	1.3 ± 2.3	1.2 ± 2.1	2.2 ± 3.8
Glucose (mg/dL)	121.1 ± 50.7	121.8 ± 49.2	115.0 ± 62.1
Lactate (mmol/L)	3.2 ± 3.0	2.9 ± 2.3	6.0 ± 5.3
BUN (mg/dL)	36.3 ± 26.1	35.1 ± 25.5	46.6 ± 28.8
Albumin (g/dL)	2.6 ± 0.7	2.7 ± 0.7	2.3 ± 0.7
Bicarbonate (mmol/L)	21.9 ± 5.9	22.2 ± 5.7	18.7 ± 6.7
Net total	−9.0 ± 1010.1	−34.3 ± 993.7	214.6 ± 1120.5
INR	1.8 ± 1.5	1.8 ± 1.4	2.3 ± 2.0
Platelet (10^3^/*μ*L)	195.6 ± 108.1	198.7 ± 107.3	167.6 ± 111.5
Age in years	65.2 ± 15.6	64.8 ± 15.8	68.7 ± 13.5

^*∗*^ *p* < 0.001 for all features except that denoted with ^*∗∗*^. ^*∗∗*^*p*=0.004.

**Table 2:** Average treatment effect (ATE) of sepsis subtypes using inverse probability weighting (IPTW) with logistic regression and T-learners machine-learning models.

	ATE	Confidence interval
*Sepsis all*		
Binarized	0.042	(0.034, 0.047)
Continuous	0.034	(0.028, 0.040)

*Pulmonary sepsis*		
Binarized	0.047	(0.037, 0.055)
Continuous	0.28	(0.22, 0.34)

*Urinary sepsis*		
Binarized	−0.013	(−0.024, −0.0035)
Continuous	−0.28	(−0.34, −0.22)

^*∗*^IPTW with a logistic regression model corresponds to binarized values. ^*∗∗*^T-learners model corresponds to continuous values.

**Table 3:** Refutation results from DoWhy library for sepsis subtypes: comparison of estimated effects with new effects and associated *P* values.

	Estimated effect	New effect	*P* value
*Sepsis all*			
Using a subset of data	0.042	0.042	0.920
Adding a random common cause	0.042	0.042	2.0

*Pulmonary sepsis*			
Using a subset of data	0.046	0.047	0.92
Adding a random common cause	0.046	0.046	2.0

*Urinary sepsis*			
Using a subset of data	−0.014	−0.014	1.0
Adding a random common cause	−0.014	−0.014	2.0

*P* values >0.05 indicate no statistically significant difference between original and refutation ATE estimates, supporting the validity of the original estimates.

## Data Availability

Data are open source and available through the eICU database.
